# Bioengineering modification and application of bacterial outer membrane vesicles

**DOI:** 10.7150/ijms.116432

**Published:** 2026-01-01

**Authors:** Yedu Wen, Yidi Si, Xinni Jia, Zhongyu Han, Zihe Zhou, Zhenchao Wu, JiaJia Zheng

**Affiliations:** 1 Department of Laboratory Medicine, Peking University Third Hospital, Beijing 100191, China.; 2 Department of Pulmonary and Critical Care Medicine, Peking University Third Hospital, Beijing 100191, China.

**Keywords:** OMVs, bioengineering modifications, clinical applications

## Abstract

Outer Membrane Vesicles (OMVs) are spherical nanovesicles naturally secreted by Gram-negative bacteria, playing key roles in nutrient uptake, toxin delivery, and the transmission of drug resistance. Recent studies have increasingly focused on the clinical potential of OMVs. Due to their remarkable biocompatibility and immunogenic properties, OMVs offer wide-ranging applications in vaccine development and antigen/drug delivery, showing great promise in the treatment of tumors, autoimmune diseases, and infections. However, challenges remain in standardizing the production and modification of OMVs, limiting their broader application. This review consolidates research on OMV modification and application, aiming to provide valuable insights to advance the development of OMV-based therapeutic strategies and clinical implementations.

## 1. General introduction to OMVs

### 1.1 The biogenesis of OMVs

Outer Membrane Vesicles (OMVs) are spherical nanovesicles naturally derived from Gram-negative bacteria, with sizes ranging from 20 to 250 nm. Their biogenesis is a complex and multifactorial process, and no single or universal mechanism fully explains their production. Existing studies suggest that OMV production can be divided into two primary categories: one involves vesicle formation through outer membrane budding in living cells, and the other arises from the aggregation of membrane fragments after cell lysis, which subsequently form OMVs. The former involves several biogenesis mechanisms, including the reduction of localized outer membrane-peptidoglycan junctions, the accumulation of periplasmic contents, and increased outer membrane curvature.

The formation of OMVs is closely linked to the interaction between the outer membrane and the peptidoglycan layer. Under normal conditions, the outer membrane and peptidoglycan are connected by proteins such as Braun's lipoprotein (Lpp), outer membrane protein A (OmpA), and components of the Tol-Pal complex, which maintain membrane stability^1^]^2^,^3^. When these connections are weakened or disrupted, the outer membrane bulges outward, leading to the production and release of OMVs. In addition to changes in membrane crosslinking, the accumulation of periplasmic contents also plays a pivotal role in triggering OMV formation. Peptidoglycan fragments or misfolded proteins in the periplasmic space exert pressure on the outer membrane, promoting vesicle production^4^. Moreover, increased outer membrane curvature facilitates OMV formation. The insertion of foreign signaling molecules into the outer membrane alters its charge balance, causing membrane bending, which leads to OMV formation, as explained by the Bilayer-Couple Model^5^.

Beyond vesicles from living bacteria, OMVs have also been identified in dead bacteria. A study by Cynthia B. *et al.* demonstrated that explosive cytolysis contributes to OMV formation. In their study, DNA damage in *Pseudomonas aeruginosa* induced the expression of phage-encoded endolysin, which degrades the peptidoglycan cell wall. This degradation causes cell rupture, and the resulting membrane fragments aggregate and assemble into OMVs^6^.

### 1.2 The composition and function of OMVs

OMVs are loaded with a diverse array of substances, including outer membrane proteins, periplasmic proteins, cytoplasmic proteins, lipopolysaccharides (LPS), phospholipids, peptidoglycan, nucleic acids, and virulence factors, with proteins and phospholipids being key components. MS-based proteomic analysis of OMVs from Gram-negative bacteria has identified hundreds of vesicle proteins^7^. The protein composition of OMVs is influenced by various factors. For instance, OMVs released by different biogenesis mechanisms in *P. aeruginosa* exhibit significant variations in protein composition^8^. The protein composition of OMVs from *Helicobacter pylori* also varies markedly under different pH growth conditions^9^. Phospholipids are key structural components of OMVs, and their types and proportions differ across bacterial species. OMVs from *Escherichia coli* ETEC are enriched in phosphatidylethanolamine (PE), phosphatidylglycerol (PG), and cardiolipin (CL), while OMVs from Actinomycetes contain high concentrations of CL and PE, *P. aeruginosa* OMVs contain predominantly PG, and *Haemophilus influenzae* OMVs are mainly composed of PE^10^.

OMVs are functionally diverse and play significant roles in nutrient uptake, stress response, biofilm formation, toxin delivery, and modulation of the host immune response. Firstly, OMVs carry hydrolytic enzymes and receptors that facilitate nutrient uptake. A study by Elhenawy *et al.* demonstrated that acid glycosidases and proteases are preferentially packaged into OMVs from Bacteroides, enhancing nutrient acquisition for the bacterial community^11^. Similarly, Ganeshwari Dhurve *et al.* found that OMVs from *Acinetobacter baumannii* DS002 were enriched in TonB-dependent transporter proteins (TonR), which are involved in the transport of iron complexes, including catecholates, hydroxamates, and hybrid iron carriers^12^. Secondly, vesiculation serves as a periplasmic stress response, where the release of OMVs is increased in reaction to stressors, thereby aiding bacterial survival in hostile environments. OMVs also play a vital role in biofilm formation. For example, *Pseudomonas* quinolone signals (PQS) released by *P. aeruginosa* are packaged within OMVs, which regulate biofilm formation and structure^13^. Furthermore, OMVs carry various virulence factors, including β-lactamase, alkaline phosphatase, lysophospholipase C, and Cycle-inhibiting factors (Cifs), which help bacteria invade host cells and modulate the host immune response, thereby promoting bacterial survival and replication^14^.

### 1.3 The biological properties of OMVs

Compared to traditional drug carriers, OMVs offer excellent biocompatibility and stability, which help maintain drug stability and reduce leakage during long-distance delivery *in vivo*. Eilien Schulz and colleagues demonstrated that OMVs are biocompatible with epithelial cells and differentiated macrophages, maintaining inherent stability under storage conditions at 4 °C, -20 °C, -80 °C, and in freeze-dried form^15^. Additionally, OMVs possess adhesins on their surface, allowing them to be recognized and endocytosed by specific cells without the need for external targeting ligands. For example, OMVs from Salmonella and Shigella contain adhesins that facilitate their recognition and endocytosis by gastrointestinal cells. This inherent targeting ability provides a natural advantage in drug delivery, particularly to specific tissues or cell types. OMVs can efficiently deliver drugs to the target site, enhancing therapeutic efficacy and reducing side effects. Another notable feature of OMVs is their immunogenicity. OMVs are rich in Pathogen-Associated Molecular Patterns (PAMPs), including LPS, peptidoglycans, lipoproteins, and flagella, which strongly stimulate the innate immune system, promoting antigen presentation and T-cell activation. The nanoscale size of OMVs further facilitates their recognition and uptake by antigen-presenting cells (APCs)^16^.

## 2. The bioengineering of OMVs

OMVs, derived from their parent cells, are rich in proteins, nucleic acids, and lipids, exhibiting exceptional biocompatibility, membrane stability, immunogenicity, safety, permeability, and tumor-targeting capabilities. Extensive research has affirmed the safety, stability, and permeability of OMVs, highlighting their potential in bioengineering. To harness OMVs for clinical applications, innovative modification strategies have been developed, mainly focusing on altering their cargo and membrane composition to enhance immunogenicity and tumor-targeting properties^18^. Numerous therapeutic and modification platforms based on OMVs have been established, showing their versatility in treating cancer, autoimmune diseases, and infectious diseases. Both modified and naturally occurring OMVs hold significant clinical therapeutic value^19^. OMV engineering strategies can be broadly categorized into three areas: gene editing, functional molecule loading, and surface modification.

### 2.1 Genetic engineering of OMVs

Genetic engineering primarily involves modifying the parental bacteria to program OMVs. Some studies have employed gene silencing through small interfering RNA (siRNA) in both *in vivo* and *in vitro* settings, or used CRISPR/Cas9-mediated gene knockout to reduce or eliminate specific OMV components^20^,^21^. Moreover, since OMVs can be secreted even under conditions of outer membrane protein deficiency, knocking out the *ompA* gene, which encodes outer membrane proteins, can significantly enhance OMV yield. These modified OMVs show improved electron transfer efficiency in Fe(III) reduction, dye degradation, and bioelectrochemical system (BES) current generation, potentially facilitating the incorporation of cargo beneficial for electron transfer and biofilm formation, such as c-type cytochromes, functional proteins, eDNA, polysaccharides, and signaling molecules^22^.

Conversely, the overexpression or introduction of certain genes can bestow OMVs with new immunogenic properties, enhance their targeting capabilities, or enable the loading of specific functional substances. For example, genetically programmed OMVs with surface modifications, such as the insertion of the extracellular domain of programmed death 1 (PD1), represent a novel and effective immunotherapeutic agent. This genetic modification does not compromise OMVs' ability to trigger immune activation. Moreover, engineered OMV-PD1 can bind to PD-L1 on tumor cell surfaces, promoting internalization and degradation, thereby protecting T cells from the PD1/PD-L1 immune suppression axis^23^.

### 2.2 Functional molecular loading of OMVs

OMVs, with their superior biocompatibility and targeting capabilities, have been utilized as carriers and vaccines. By loading functional molecules such as enzymes, antibiotics, and nucleic acids, and exploiting the exceptional targeting properties of OMVs, it is possible to regulate cellular activities and the survival environment. OMVs derived from attenuated Klebsiella pneumoniae or *E. coli* are commonly used for tumor drug loading. These OMVs not only act as biological nanocarriers for chemotherapeutic agents but also elicit appropriate immune responses, making them promising candidates for tumor chemoimmunotherapy^24^,^31^.

Current drug-loading methods include incubation, extrusion, and sonication^26^,^27^. However, antibiotic loading is more distinctive. Given that bacteria can eliminate antibiotics by secreting OMVs—a mechanism contributing to antimicrobial drug resistance—antibiotics may be encapsulated within OMVs when bacteria secrete them in the presence of antibiotics^28^. Additionally, due to the phospholipid composition of OMV membranes, liposome wrapping has been used to transport hydrophilic drugs like doxorubicin (DOX) into OMVs^29^,^30^. As natural nanocarriers, OMVs possess a mechanically stable bilayer structure, conferring excellent biostability. Antibiotic-loaded OMVs retain most of the native adjuvant components derived from their parent bacteria. These components promote immune maturation, induce controlled inflammatory responses, and enhance the immunogenicity of the tumor microenvironment. This mechanism not only recruits macrophages but may also synergize with the encapsulated antibiotics^30^,^31^.

OMVs loaded with nucleic acids hold significant potential for various applications, offering enhanced safety and substantial cargo capacity, making them effective genetic engineering tools^32^. Current bacterial modification methods for nucleic acid delivery include engineering attenuated strains, lysis circuits, and conjugation mechanisms^33^,^34^,^35^. OMVs can also benefit the development of autonomous nanorobots, which are advanced precision therapeutic tools. Traditional nanorobot designs rely primarily on inorganic materials, which often have poor biocompatibility and limited biological functionality. This has prompted exploration of OMVs in nanorobotics^36^. Nanorobots surface-engineered with cell-penetrating peptides leverage OMV membrane properties to promote tumor targeting and penetration, effectively protecting the loaded gene-silencing tool siRNA from enzymatic degradation, thus enhancing siRNA delivery and immune stimulation^37^.

Despite current advancements, the drug-loading concentration in OMVs remains insufficient due to the barrier posed by the vesicle membrane. Additionally, the lack of standardized clinical production and enrichment methods for OMVs limits their broader application. While safety has largely been validated, OMVs derived from pathogenic bacteria may still contain residual endotoxins and other harmful components that are difficult to remove, potentially leading to side effects in clinical applications^28^. Further research is needed to develop effective, standardized methods for OMV production, enrichment, and endotoxin removal.

### 2.3 Surface functionalization of OMVs

The surface of OMVs exhibits exceptional plasticity, and modifications can enhance their biocompatibility and permeability. For instance, Li-Hua Peng *et al.* employed an αvβ3 integrin-targeting ligand and indocyanine green to modify OMVs derived from genetically engineered *E. coli*, demonstrating superior penetration of the stratum corneum and increased specificity toward melanoma^38^. OMVs also possess notable immunogenicity, with the specific presentation of antigens on their surface capable of triggering targeted anti-tumor immune responses, making them valuable for cancer treatment. The potential of genetically modified OMVs, such as those with the extracellular domain of PD1 inserted, has been successfully validated^23^,^39^,^40^. Recently, Keman Cheng *et al.* developed a multifunctional OMV-based platform, displaying tumor antigens on the OMV surface by fusing them with ClyA protein. This process, streamlined by a Plug-and-Design system using a tag/capture protein pair, enables OMVs modified with various protein capture agents to simultaneously display multiple tumor antigens, eliciting synergistic anti-tumor immune responses^41^. Additionally, the surface charge of OMVs plays a critical role in their modification. One study revealed that the activation of immune responses by OMVs is inversely related to their surface positive charge, which has important implications for OMV surface engineering^42^.

Surface modification presents a promising solution to many challenges limiting the clinical translation of OMVs. Protein expression bottlenecks and difficulties in localizing antigens to the host cell's outer membrane can make antigen display on OMVs unpredictable, particularly for bulky or complex antigens, significantly reducing the efficacy of OMV-based vaccines. To address this, a study proposed biotinylating surface antigens on OMVs. Kevin B. Weyant and colleagues developed a modular platform to link biotinylated antigens to the OMV exterior. They demonstrated that OMVs could be easily modified with a wide range of biotinylated subunit antigens, including globular and membrane proteins, glycans, glycoconjugates, haptens, lipids, and short peptides. When these OMV formulations were injected into mice, a robust antigen-specific antibody response was observed, highlighting a significant improvement in the immunogenicity and specificity of OMV-based vaccines^43^.

Systemic toxicity caused by PAMPs in OMVs is a significant barrier to their clinical translation, as the specific presentation of antigens on the OMV surface to trigger targeted anti-tumor immune responses can lead to toxic side effects. This issue can be mitigated through surface mineralization. Xue Chen *et al.* coated melanin-loaded OMVs (OMVMel) with calcium phosphate (CaP) *via* surface mineralization to create OMVMel@CaP. Compared to OMVMel, OMVMel@CaP exhibited reduced systemic inflammatory responses and less damage to the liver, spleen, lungs, and kidneys, allowing for increased dosage to enhance anti-tumor effects. In the acidic tumor microenvironment, the CaP shell decomposes, releasing OMVMel and triggering anti-tumor immune responses. The dual stimulation from OMVs as immune adjuvants and DAMPs released through photothermal effects significantly improved tumor treatment efficiency by promoting the infiltration of mature dendritic cells (DCs), M1 macrophages, and activated CD8^+^ T cells, while reducing the proportion of myeloid-derived suppressor cells (MDSCs) in tumors^44^. Similarly, anchoring OMVs with Fe^2+^ through electrostatic interactions and loading them with the STING agonist-4, followed by tumor-targeting DSPE-PEG-FA modification, functionalized the OMVs. This approach enhanced tumor-targeting capabilities, achieved tumor-specific therapy, and minimized side effects^45^,^46^.

Surface modification of OMVs has been widely applied in the development of personalized tumor vaccines, enhancing both their safety and immunogenicity. However, surface-modified OMVs still face challenges in clinical applications due to the lack of standardized production methods, limited effectiveness, and high modification costs. To fully realize their clinical potential, significant obstacles related to efficacy and large-scale production must be addressed^47^,^48^,^49^. Lipid nanoparticles and synthetic liposomes (including modern LNPs for mRNA delivery) are highly tunable in composition, surface chemistry, and cargo loading, with well-established, scalable GMP manufacturing routes that ensure reproducible stability, controlled pharmacokinetics, and reduced innate immune activation unless deliberately engineered otherwise. Hybrid strategies that combine OMV-like biological cues with the tunability and stability of synthetic lipid platforms are emerging as a promising approach to capture the benefits of both systems^50^,^51^,^52^.

### 2.4 Auxiliary modification of OMVs

Fusion culture is an emerging specialized cultivation method that combines OMVs with other biological entities and products to enhance their tumor immune effects. Current research has primarily focused on oncolytic viruses. Oncolytic viruses cultivated in fusion with mineralized OMVs have demonstrated prolonged drug circulation times and enhanced immune responses in mouse models^53^,^54^. However, this technology requires higher viral titers, significantly increasing the cost and complexity of modification. Therefore, future research should concentrate on downstream processing techniques for oncolytic viruses and purification methods for OMVs to improve their clinical applicability^55^. Macrophage membrane vesicles (MMVs) represent another promising material for integrated cultivation. MMVs not only synergize with OMVs to induce a robust immune response but also neutralize endotoxins in OMVs, enhancing their safety. This approach has shown favorable therapeutic outcomes in a mouse model of sepsis caused by *P. aeruginosa* infection. Further experiments with human-derived macrophages are necessary to validate its safety and efficacy^56^.

Moreover, cultivation under specific environments can modulate the properties of OMVs by influencing gene expression. One study found that serum-induced OMVs in the mosquito gut contained serum-derived lipids, such as phosphatidylcholine. These OMVs selectively targeted parasites, rendering mosquitoes resistant to Plasmodium infection, which offers a potential strategy to block malaria transmission^57^. Another study demonstrated that OMVs released by *Akkermansia muciniphila* in the human gut, trained by garlic ELNs (GaELNs), could reverse high-fat diet-induced type 2 diabetes mellitus (T2DM) in mice. This reveals a molecular mechanism where OMVs from gut bacteria trained by plant nanoparticles regulate brain gene expression, influencing the treatment of brain dysfunction caused by metabolic syndrome^58^. Similarly, the highly active *A. baumannii* phage lysin LysP53 can stimulate OMV production after interacting with *A. baumannii*, *E. coli*, and Salmonella. OMVs produced in this manner exhibited higher protein concentrations and lower endotoxin levels, resulting in better therapeutic effects in a mouse model of pneumonia^59^.

Nanoparticleization of OMVs is another promising strategy. The most critical factor in inducing an effective immune response is the uptake of antigen particles by APCs^60^. The absorption of bacterial membrane vesicle particles by APCs is size-dependent. Smaller vesicle particles are more efficiently absorbed by APCs, while larger particles can present more antigens. By optimizing the size of bacterial membrane vesicles, the immune response can be maximized^61^,^62^. Therefore, achieving nanoparticleization of OMVs is a worthwhile supplementary modification strategy.

While genetic engineering, functional molecule loading, and surface functionalization remain the primary methods for modifying OMVs, auxiliary strategies such as fusion culture, cultivation under special environments, and nanoparticleization are essential for enhancing OMV properties. These approaches are essential for advancing OMV-based biomedical applications and overcoming the current limitations in clinical translation.

## 3. Application of OMVs

OMVs, due to their significant biological relevance and application potential, have garnered widespread attention in recent years, particularly in the fields of anti-infection, oncology, and autoinflammation. OMV research not only provides new therapeutic approaches for these diseases but also offers valuable insights into various physiological and pathological processes, holding promising prospects in the medical field.

### 3.1 Anti-infective therapy

OMVs have shown considerable promise in combating infections, particularly in inhibiting pathogenic microorganisms. They exert their antimicrobial effects through multiple mechanisms, effectively suppressing the growth and spread of pathogens (Table 1).

OMVs can directly interact with pathogenic microorganisms, disrupting their normal physiological and metabolic processes^63^,^64^. Inter-microbial competition often occurs *via* OMVs, which contain specific components capable of binding to surface receptors of pathogens or being internalized by them. This interaction can block nutrient uptake or signal transduction pathways, thereby inhibiting pathogen growth and reproduction^65^. For instance, OMVs enriched with phosphatidylcholine selectively target Plasmodium and deliver effector proteins to kill the parasite, offering a novel strategy for malaria treatment^57^. The OMV protein PA022 from *P. aeruginosa* inhibits the growth of *A. baumannii* strains, suggesting that PA022-containing OMVs could serve as a viable alternative for mitigating *A. baumannii* infections^66^. Furthermore, *P. aeruginosa* PAO1 produces OMVs carrying a 26-kDa β-glycosidase (autolysin), which exhibits broad-spectrum antibacterial activity, providing a promising avenue for developing new therapeutic strategies^67^,^68^,^69^. OMVs derived from *E. coli* have been utilized as highly selective antimicrobial agents to create stable implant coatings. These OMV coatings exhibit differential therapeutic effects, curing wounds infected with heterologous bacteria while exacerbating those infected with homologous bacteria^70^. However, naturally derived OMVs present significant limitations for anti-infective applications. Specifically, OMVs from certain bacterial species exhibit activity only against closely related bacteria, and the presence of virulence factors and antibiotic resistance genes they carry may potentially exacerbate host infections.

OMVs also exhibit potent immunostimulatory properties, capable of inducing host immune responses^72^. Their surfaces carry various PAMPs, such as LPS, peptidoglycan, and lipoproteins, which are recognized by pattern recognition receptors (PRRs). This recognition activates the innate immune system, promoting the activation of immune cells such as macrophages and DCs, as well as the secretion of cytokines, thereby enhancing the host's ability to combat pathogens. This mechanism underlies the current use of OMVs in anti-infective therapies^73^,^74^,^75^.

Several OMV-based strategies have been developed to treat *H. pylori* infections. OMVs derived from *H. pylori* cultured under specific conditions or with virulence genes (e.g., CagA, VacA, DupA) deleted exhibit reduced virulence factor content while retaining immunostimulatory capacity, making them effective and safe vaccine candidates^20^]^21^,^73^. Similarly, OMVs from *Burkholderia pseudomallei* grown under macrophage-mimicking conditions have been shown to provide significant protection against pulmonary infections in mice, comparable to live attenuated vaccines. The second-generation M9 OMV vaccine, with its low toxicity, strong adjuvant properties, and immunogenicity, is considered a promising candidate for further development^74^. Furthermore, a case-control study on Neisseria meningitidis serogroup B vaccination found lower incidences of meningitis and gonorrhea in OMV vaccine recipients compared to non-OMV vaccinees, highlighting the potential advantages of OMV-based vaccines in reducing disease incidence^77^. In summary, OMVs function as potent immune activators with considerable promise for vaccine and adjuvant development.

In addition to their immunomodulatory roles, OMVs serve as effective drug delivery vehicles, enabling targeted delivery of antimicrobial agents to infection sites. With the rise of antibiotic resistance due to misuse, OMVs have emerged as a promising biomaterial for antibiotic delivery^78^,^79^,^80^. For example, gentamicin-loaded OMVs from *P. aeruginosa* PAO1 exhibit potent antibacterial activity against Staphylococcus aureus and other pathogens, as they contain both autolysin and small amounts of gentamicin, which act synergistically^81^. Similarly, *A. baumannii* produces OMVs carrying antibiotics such as amikacin and ciprofloxacin through efflux mechanisms. Mouse studies have shown that OMV-mediated antibiotic delivery not only increases local drug concentrations but also reduces systemic exposure, significantly improving safety and efficacy^67^,^82^. However, not all antibiotic-loaded OMVs enhance antimicrobial activity. Some may inadvertently promote resistance. For instance, Marie Burt *et al.* found that OMVs from Klebsiella pneumoniae bound to polymyxin acted as decoys, preventing antibiotic interaction with bacterial surfaces and thereby protecting the bacteria^83^. In summary, OMV-based therapies offer unique advantages for targeted antibiotic delivery due to their exceptional stability and biocompatibility. While OMV-based therapies show substantial promise for anti-infective applications, critical challenges remain that must be resolved before clinical translation.

### 3.2 Anti-tumor therapy

In recent years, OMVs have shown great promise in anti-tumor therapy, as they can suppress tumor initiation and progression through multiple mechanisms (Table 2).

OMVs can directly act on tumor cells and induce their apoptosis. The enrichment of PAMPs enables OMVs to trigger pyroptosis or apoptosis at the cellular level *via* receptor-activated death pathways. A recent study found that the OMVs of *Fusobacterium nucleatum* (Fn-OMV) can upregulate the expression of PANoptosis execution proteins gasdermin D/E (GSDMD/E) and mixed lineage kinase domain-like protein (MLKL) by interfering with the ubiquitination process^53^. Yao Jiang *et al.* discovered that OMVs derived from *E. coli* reduce the ratio of the anti-apoptotic protein Bcl-2 to the pro-apoptotic protein Bax, thereby inducing apoptosis in colon cancer cells (CT26) and inhibiting the progression of colon cancer^84^. Consistent with *in vitro* findings, significant reductions in tumor volume and weight were observed in murine models. However, cells can counteract OMV-induced cell death through multiple pathways, limiting the therapeutic efficacy of OMVs when used alone. For practical applications, modification and engineering of OMVs are required to overcome these limitations.

OMVs can also modify the tumor microenvironment, inhibiting tumor growth, invasion, and metastasis, and enhancing the efficacy of tumor treatments. The tumor microenvironment is a specialized ecosystem created by tumor cells, encompassing immune and inflammatory cells, tumor-associated fibroblasts, adjacent stromal tissues, tumor blood vessels, and various cytokines and chemokines. This environment can facilitate tumor growth, metastasis, and diffusion, while interfering with the efficacy of tumor therapies^85^,^86^. Engineered OMVs can reprogram the tumor microenvironment, improving anti-tumor effects. For example, engineered OMVs-PD1 can comprehensively regulate the tumor microenvironment. On one hand, OMVs activate the immune response within the host due to their PAMPs, inducing strong expression of IFN-γ and T cell-mediated anti-tumor effects. On the other hand, the programmed death receptor 1 (PD-1) inserted on the surface of OMVs binds to tumor cells, blocking their inhibition of T cell proliferation and promoting tumor cell apoptosis^87^. Shuang Qing *et al.* covered OMVs with a pH-sensitive CaP shell, which helps macrophages polarize from M2 to M1 and enhances the expression of tumor suppressor genes. Additionally, the outer shell of OMVs can be functionalized with components such as folic acid to promote targeted tumor therapy^88^. In summary, the inherent immunogenicity and novel properties conferred by engineering modifications enable OMVs to reprogram the tumor microenvironment, enhancing their anti-tumor effects.

OMVs can also serve as nanoparticle carriers to precisely deliver anti-tumor active substances to tumor sites. Ze Mi *et al.* Loaded DOX into Salmonella-derived OMVs to construct OMVs/DOX. By leveraging the ability of Salmonella to selectively colonize hypoxic tumor sites and the selective recognition of OMVs/DOX by neutrophils, they significantly enhanced the therapeutic effect on glioma^89^. OMVs carrying perhexiline also exhibit favorable anti-tumor effects^90^. Oncolytic adenovirus (Ad) infection can promote tumor cell autophagy to exert anti-tumor effects. A study encapsulated Ads in OMVs and covered them with a biomineral shell to promote their accumulation in tumors, thereby achieving enhanced autophagic cascade anti-tumor immunity^91^. Vipul Gujrati *et al.* engineered *E. coli* to carry siRNA targeting kinesin spindle protein (KSP) with an affinity for human epidermal growth factor receptor 2 (HER2), successfully inhibiting the growth of breast cancer cells^92^. The nanoscale dimensions of OMVs confer potent tissue penetration capabilities, facilitating their use as effective drug delivery vehicles for targeted anti-tumor therapy.

Furthermore, OMVs are frequently employed as delivery vehicles for tumor antigens and as adjuvants to enhance tumor therapeutic outcomes. Due to the marked interpatient heterogeneity of tumor cells, conventional vaccine formulations exhibit limited efficacy across diverse cases, highlighting the need for personalized vaccine strategies. To address this, bacterial OMV-based nanoplatforms have emerged as customizable delivery systems capable of incorporating patient-specific tumor antigen profiles. These OMV platforms exhibit dual functionality as both antigen carriers and built-in immunostimulants, facilitating the clinical development of personalized cancer vaccines^93^. Epitopes derived from embryonic stem cells (ESCs) can serve as therapeutic tumor vaccines against various types of tumors. Meiling Jin *et al.* utilized OMVs to deliver ESC-derived tumor antigens and immune checkpoint inhibitors (PD-L1 antibodies), effectively inhibiting tumor growth^94^. Additionally, a study developed an oral tumor vaccine based on OMVs. Yale Yue *et al.* genetically engineered *E. coli* to produce OMVs carrying tumor antigens in situ within the intestine. These OMVs then traversed the intestinal epithelial barrier and were recognized by immune cells in the lamina propria, effectively activating tumor antigen-specific immune responses and significantly suppressing tumor growth^95^. OMVs can also be employed for therapeutic mRNA vaccination. Yao Li *et al.* added RNA-binding protein L7Ae and lysosomal escape protein listeriolysin O to the surface of OMVs (OMV-LL). OMV-LL bound to mRNA antigens through L7Ae and delivered them to DCs, achieving cross-presentation through listeriolysin O-mediated endosomal escape, which significantly inhibited melanoma progression^96^. In summary, OMVs—acting as nanocarriers with intrinsic immune adjuvant properties—are widely utilized in tumor vaccine research to deliver tumor antigens and elicit robust antigen-specific anti-tumor immune responses.

### 3.3 Autoimmune and inflammatory diseases

The inflammatory response is a complex physiological process triggered by injury and infection, but excessive or prolonged inflammation can contribute to the onset and progression of autoinflammatory diseases. Autoimmune diseases occur when the body's immune system mistakenly attacks its own healthy tissue cells. Due to the diverse and complex mechanisms underlying their development, autoimmune diseases encompass a wide range of conditions that can involve multiple systems and organs, leading to significant health issues. OMVs can modulate autoimmune and inflammatory diseases through various mechanisms (Table 3).

In inflammatory diseases, OMVs primarily function through three mechanisms. First, OMVs regulate cell proliferation, differentiation, and cytokine secretion by interacting with receptors on the surface of immune cells^99^. For example, Durant *et al.* found that during *in vitro* culture of DCs, Polysaccharide A expressed on the surface of *Bacteroides tenuis* OMVs promoted the production of regulatory T cell (Tregs) responses and interleukin-10 by DCs in a *TLR-2* and *GADD45α*-dependent manner, showing beneficial effects in a mouse model of acute colitis^99^. Additionally, Chu *et al.* conducted a study using Atg16l1fl/fl Cd11cCre mice, demonstrating that B. tenuis OMVs can activate LC3-related phagocytosis (LAP) *via* the *ATG16L1* gene, acting on TLRs and inducing Tregs to inhibit mucosal inflammation, thereby offering protection in colitis^101^.

Additionally, OMVs have a regulatory effect on cell signaling pathways, influencing the cellular response to various environmental and situational factors. They can interfere with intracellular signaling pathways, such as the NF-κB and MAPK pathways^76^. For instance, Zhang *et al.* demonstrated that Fn-OMVs enter human periodontal ligament stem cells (hPDLSCs) primarily through fosprotein-dependent endocytosis, activating NF-κB signaling and impairing osteogenic differentiation. Furthermore, in a periodontitis mouse model, OMVs were found to facilitate signal transduction *via* the NF-κB pathway, leading to the activation of inflammasomes and the release of IL-1β and IL-18^97^.

OMVs predominantly function by regulating inflammatory factors and can effectively activate inflammasome pathways, particularly those mediated by NLRP3 and Caspase-11/4. For example, Chen *et al.* investigated streptozotocin-induced diabetic rats and found that fecal bacterial OMVs (fBEVs) significantly upregulated the expression of pro-caspase-11, active caspase-11, pro-caspase-1, and active caspase-1 in renal tissue. Similarly, in HK-2 cells, fBEVs activated caspase-4 (a human homolog of caspase-11), and silencing caspase-4 with siRNA inhibited this process^96^. Zhang *et al.* established a rat model of periodontitis and found that Fn-OMVs triggered the activation of NLRP3 inflammasomes in hPDLSCs and the subsequent release of IL-1β and IL-18, resulting in impaired mineralization in these cells^97^. Moreover, compared to wild-type (WT) cells, OMV stimulation increased the transcription of multiple pro-inflammatory cytokines in Atg16L1^ΔCD11c^ DCs, which are key complexes involved in the inflammatory response^101^]^107^.

For autoimmune diseases, OMVs function through a similar mechanism. They may promote the production and function of Tregs, thereby leading to autoimmune responses^100^,^102^. This principle can be applied to regulate the quantity and expression of immune cells, suppressing excessive immune responses. OMVs may also stimulate the immune system through signaling pathways, triggering corresponding responses. For instance, OMVs can regulate toll-like receptor-mediated signaling pathways to inhibit the inflammatory response in patients with systemic lupus erythematosus (SLE)^76^. Additionally, OMVs may influence the autoimmune process by affecting the activation and regulation of cytokines. Chen *et al.* conducted a comprehensive study and found that OMVs can regulate immune cells and cytokines in various ways, a response confirmed in multiple autoimmune disease models, including lupus erythematosus, multiple sclerosis, and rheumatoid arthritis^106^.

OMVs also target autoimmune and inflammatory diseases through various mechanisms, such as reducing autoimmune responses, promoting immune tolerance, and enhancing the self-repair ability of the immune system. This may occur through simulating pathogen infection or providing specific immune stimulation signals. OMVs can promote immune system tolerance to self-antigens^58^,^104^. In the study of myasthenia gravis, patients were induced to develop immune tolerance through oral tolerance treatment strategies, leading to a reduction in disease symptoms^104^. By training the immune system, OMVs may enhance its ability to repair itself, improving its capacity to respond to autoimmune disease challenges. This enhancement may involve aspects such as the proliferation, differentiation, and functional improvement of immune cells^99^.

OMVs, based on these mechanisms, have broad therapeutic applications. They can serve as diagnostic biomarkers; for instance, elevated levels of OMVs derived from the gut microbiota are associated with the severity of diabetic nephropathy^96^. Similarly, OMV-related proteins in periodontitis can indicate disease activity^97^. Given their immune characteristics, OMVs can also be developed as a vaccine platform for treating inflammatory diseases or symptoms. In 2015, the success of the Neisseria meningitidis OMV-based vaccine laid the foundation for developing similar strategies against other pathogens^98^. Additionally, OMVs naturally possess the ability to cross biological barriers and target specific cells, making them attractive drug delivery carriers. Engineered OMVs are currently used to deliver therapeutic agents to inflammatory sites while minimizing systemic side effects^102^. Furthermore, regulating the microbiome, particularly targeting the gut microbiota, may offer new approaches to treating related diseases. Fecal microbiota transplantation (FMT) shows promise in restoring normal OMV profiles and reducing inflammation in diabetic models^96^.

Despite the great potential of OMVs in treating inflammatory and autoimmune diseases, several challenges remain. Although OMVs activate the immune system through various mechanisms, the inflammatory responses they trigger can negatively impact treatment. OMVs from *H. pylori* induce local and systemic inflammatory responses by releasing pro-inflammatory factors like LPS, which may result in serious consequences, such as gastric mucosal barrier dysfunction and gastric cancer^105^. Moreover, OMVs can exacerbate disease pathology by activating autoimmunity. For example, *H. pylori* OMVs may trigger an autoimmune response through components similar to host blood group antigens^105^,^107^. Recent studies have also shown that *H. pylori* OMVs are absorbed by brain astrocytes, exacerbating amyloid-β accumulation and cognitive decline through the complement component 3 (C3)-C3a receptor (C3aR) signaling pathway, accelerating the development of Alzheimer's disease^108^[109. Additionally, the toxicity of OMVs and the limitations of immature production and purification technologies remain major barriers to their application.

## 4. Conclusion

The application of OMVs in anti-infection, tumor therapy, and autoinflammation is highly significant. While progress has been made through exploration in various fields, many issues still need to be further studied and resolved.

### 4.1 Current Status

As a novel biomaterial, OMVs have made significant progress in bioengineering. Gene editing, functional molecular loading, and surface functionalization for OMVs have largely been realized, but further research efforts are still required to bridge the gap from basic to clinical applications. In the realm of anti-infection, OMVs have shown promise in inhibiting pathogenic microorganisms and serving as vaccine adjuvants, offering new approaches for the prevention and treatment of infectious diseases. However, the mechanisms of action need further clarification to optimize their application and minimize potential risks. In cancer therapy, OMVs' effects on tumor cells and their role in tumor immunotherapy indicate their potential in cancer treatment. However, to achieve broader and more effective clinical applications, the heterogeneity of tumors must be addressed. In autoinflammatory diseases, OMVs' regulatory effects on the inflammatory response and their therapeutic potential provide new hope for treatment. However, more in-depth research is needed to clarify their specific targets and regulatory networks.

### 4.2 Future Perspectives

Despite notable advances in OMV engineering and therapeutic applications, several critical challenges must be addressed before clinical translation is feasible. First, the lack of standardized and scalable manufacturing workflows remains a major obstacle. Current OMV production relies heavily on strain-specific optimization and laboratory-scale isolation procedures, leading to batch-to-batch variability and limited reproducibility. Future efforts should focus on developing GMP-compliant large-scale fermentation, purification, and endotoxin removal processes, along with universally accepted quality control benchmarks.

Second, the safety assessment of OMVs remains insufficient. Endotoxin contamination, unintended immune hyperactivation, and incomplete characterization of OMV cargo continue to pose risks. Comprehensive toxicology, immunogenicity, and biodistribution studies in patient-derived xenograft (PDX) or humanized models are essential to support clinical translation. In parallel, synthetic biology strategies, such as LPS detoxification, lipid A remodeling, and surface deactivation, may enhance safety while preserving adjuvant efficacy.

Third, improvements in targeting efficiency and functional precision are still needed. Although surface engineering and genetic modification have enhanced selectivity, targeting performance remains heterogeneous across tumor types, inflammatory states, and infectious microenvironments. Future approaches should explore modular plug-and-play antigen-display systems, programmable targeting ligands, and microenvironment-responsive OMV designs to achieve more precise therapeutic outcomes.

Finally, integrating OMV-based platforms with other therapeutic modalities—including immune checkpoint inhibitors, oncolytic viruses, mRNA vaccines, and nanomedicines—represents a promising yet underexplored direction. Advancements in this area will require sustained interdisciplinary collaboration across microbiology, immunology, materials science, and clinical medicine to develop highly personalized and clinically deployable OMV-based therapies.

## Figures and Tables

**Figure 1:**
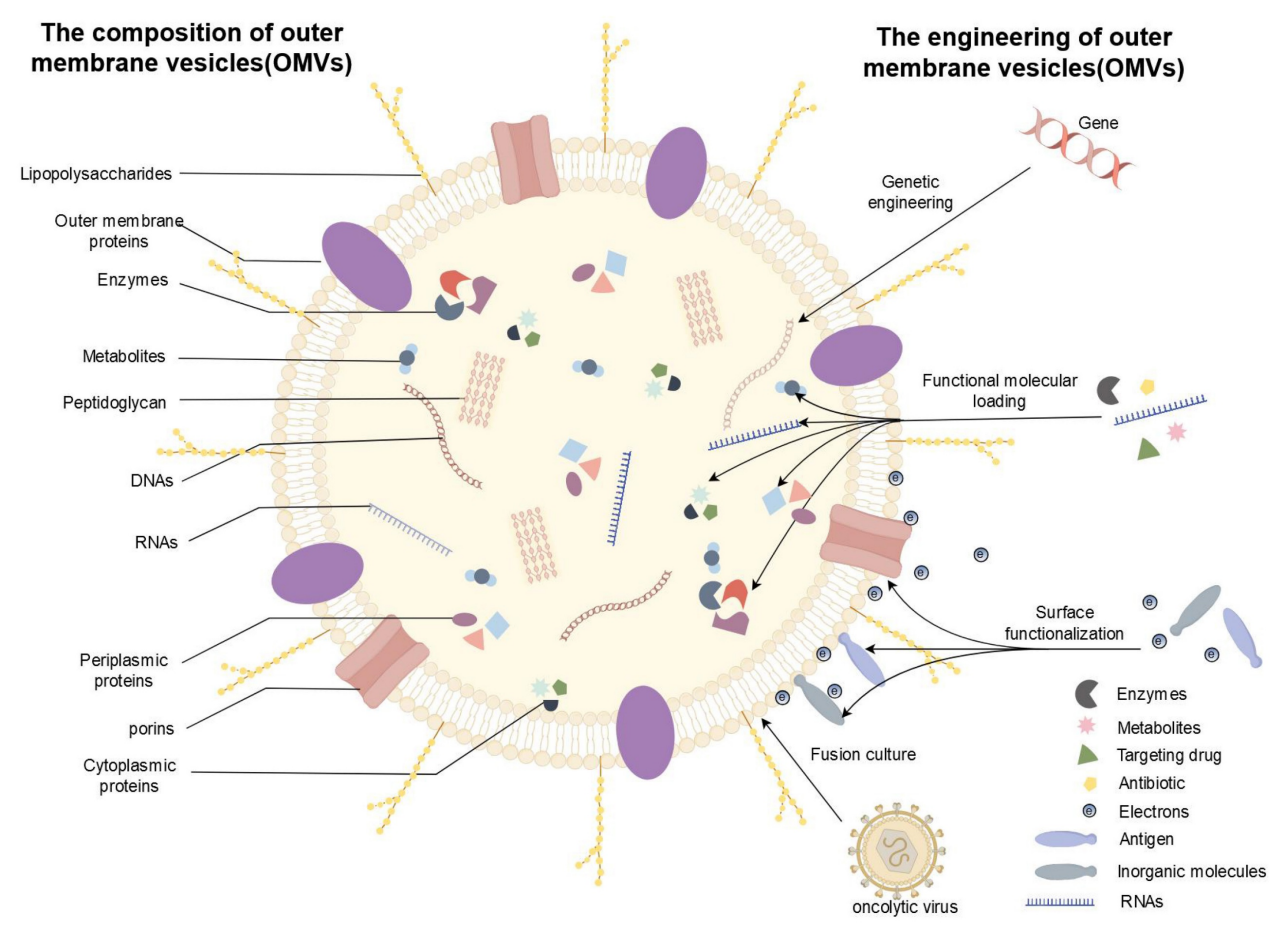
The composition and bioengineering of OMVs. OMVs, primarily composed of bacterial outer membranes, periplasmic space, and cytoplasmic content, demonstrate exceptional potential for bioengineering. (1) Genetic Engineering: The yield and components of OMVs can be modified by knocking out or introducing specific genes. (2) Functional Molecular Loading: By loading functional molecules such as enzymes, antibiotics, and nucleic acids, and leveraging the unique targeting properties of OMVs, cellular activities and the survival environment can be regulated. (3) Surface Functionalization: Surface modification using antigens, inorganic molecules, and electrons offers promising solutions to challenges hindering the clinical translation of OMVs, enhancing their biocompatibility and permeability. (4) Auxiliary Modification: Utilizing fusion culture and other auxiliary modifications to adjust the composition of OMVs plays a pivotal role in enhancing their properties. (By Figdraw).

**Figure 2:**
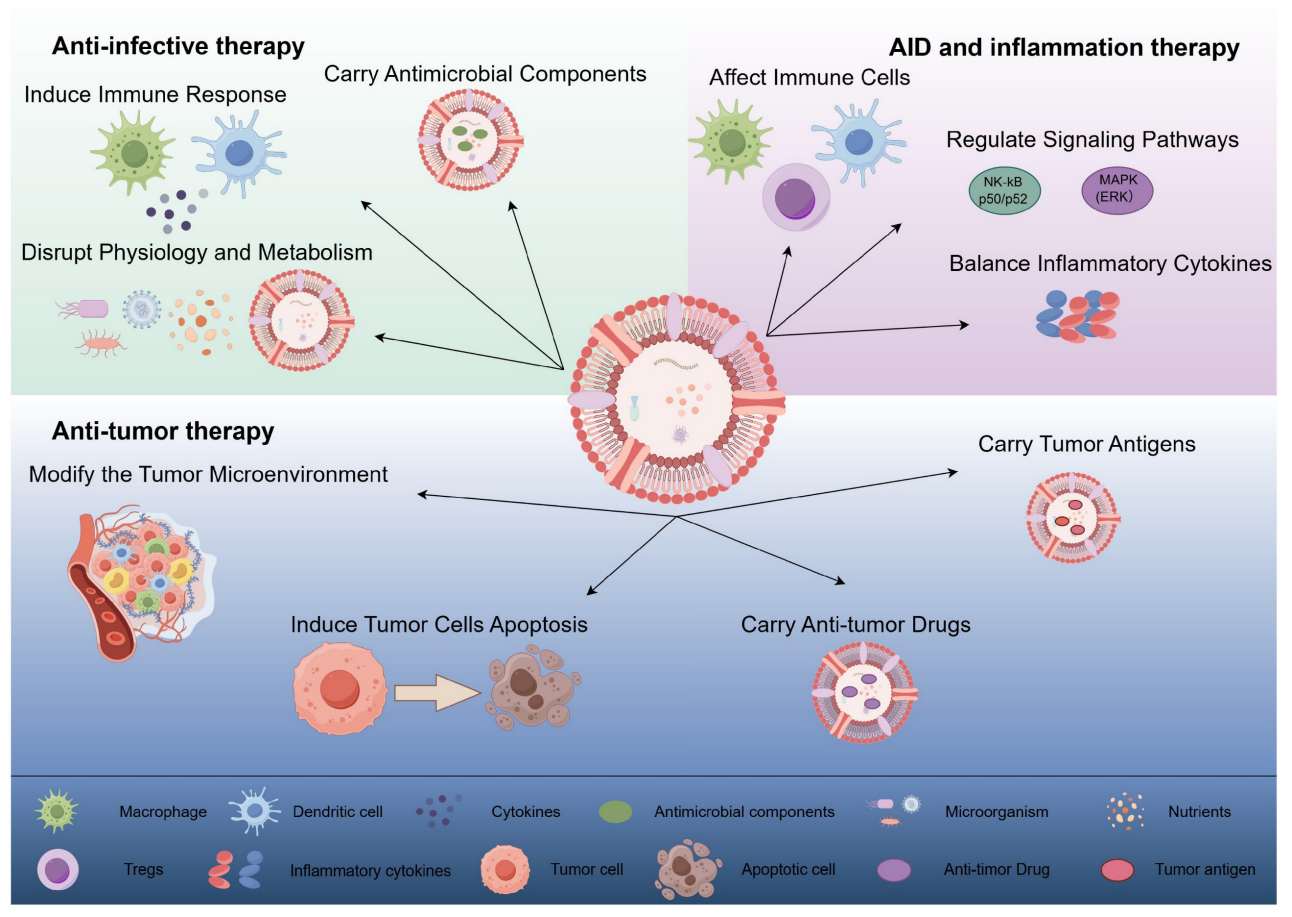
OMVs exert anti-infective, anti-tumor, and autoimmune/inflammatory effects through different mechanisms. (1) Anti-infective therapy: OMVs interfere with the normal physiological and metabolic processes of microorganisms by directly acting on them, inducing host immune responses, and carrying antimicrobial components to exert anti-infective effects. (2) Anti-tumor therapy: OMVs can directly induce apoptosis in tumor cells, modify the tumor microenvironment, and deliver anti-tumor drugs and tumor antigens to exert anti-tumor effects. (3) Autoimmune disease and inflammation therapy: OMVs contribute to the therapy of autoimmune diseases and inflammation by affecting immune cell activation and function, regulating inflammation-related signaling pathways, and promoting the balance of anti-inflammatory and pro-inflammatory cytokines. (By Figdraw).

**Table 1:** Application of OMVs in anti-infection

Application strategy	Application principles and examples	Disease model	Reference
Direct inhibition of pathogenic microorganisms	Virulence factors such as β-lactamase and alkaline phosphatase carried by OMVs inhibit the infection of *Pseudomonas aeruginosa*	*Pseudomonas aeruginosa* infection model	[14, 28
Vaccine adjuvant	OMVs act as vaccine adjuvants, binding antigens to enhance immunogenicity (e.g. binding *Neisseria meningitidis* antigens for vaccine development)	Neisseria meningitidis vaccine research	17, 88
Competitive niche	OMVs inhibit the growth of pathogenic microorganisms by occupying their living space (e.g., by suppressing Plasmodium-infected mosquito models)	Mosquito models infected with Plasmodium	53
Antibacterial activity	OMVs carry antimicrobial proteins (such as hemolytic phospholipase C) that can directly kill pathogens	*Acinetobacter baumannii* infection model	12, 67
Modulation of host immunity	OMVs activate macrophages and dendritic cells and enhance immune clearance against *H. pylori*	*Helicobacter pylori* infection model	60, 61
Genetic engineering modification	Knocking out LPS genes in OMVs (such as ΔmsbB mutants) reduces toxicity and preserves immune activation	Mouse infection model	20, 71

**Table 2:** Application of OMVs in tumor therapy

Application strategy	Application principles and examples	Disease model	Reference
Immune checkpoint suppression	Genetically engineered OMVs display the PD1 outer domain on the surface, block the PD1/PD-L1 axis, and activate the anti-tumor immune response of T cells	Melanoma model and lung cancer model	23, 40
Drug delivery	Doxorubicin-loaded OMVs target non-small cell lung cancer, enhancing chemotherapy and inducing an immune response	Non-small cell lung cancer model	23, 40
Photothermal therapy	OMV Surface Modified Indocyanine Green (ICG) kills tumor cells and releases damage-associated molecular patterns (DAMPs) through photothermal effects	Melanoma model	38, 86
Gene therapy	OMVs carry siRNA to silence tumor-related genes (such as *STAT3*) and inhibit tumor growth	Breast cancer model	37, 86
Combination therapy	OMVs, in combination with oncolytic viruses such as HSV-1, induce panapoptosis to enhance anti-tumor immunity	Colon cancer model	49
Surface mineralization technology	Melanin-loaded OMV@CaP reduces systemic toxicity through surface mineralization and enhances tumor targeting and photothermal therapy effect	Liver cancer model	42

**Table 3:** Application of OMVs in autoimmune and inflammatory diseases

Application strategy	Application principles and examples	Disease model	Reference
Adjust inflammatory signaling pathways	OMVs reduce inflammation by inhibiting NF-κB and MAPK pathways (as in rheumatoid arthritis models)	Rheumatoid arthritis models	[20, 63
Induce immune tolerance	OMVs carry autoantigens (such as SLE-associated antigens) that induce immune tolerance through oral tolerance strategies	Systemic lupus erythematosus (SLE) model	62, 102
Intestinal flora adjustment	Garlic exosome (GaELNs)-trained *Akkermansia muciniphila* releases OMVs and reverses high-fat diet-induced type 2 diabetes	Mouse model of type 2 diabetes mellitus	54
Neuroinflammatory regulation	*Helicobacter pylori* OMVs promote Alzheimer's disease neuroinflammation through the complement C3-C3aR signaling pathway	Alzheimer's disease model	105
Anti-inflammatory cytokine induction	OMVs promote the secretion of anti-inflammatory factors IL-10 and TGF-β and inhibit pro-inflammatory factors (such as TNF-α)	Inflammatory bowel disease (IBD) model	96, 98
Skin inflammation inhibition	*Parabacteroides goldsteinii*-derived OMVs reduce psoriasis inflammation by regulating skin flora	Psoriasis mouse model	102
